# When One Health Meets the United Nations Ocean Decade: Global Agendas as a Pathway to Promote Collaborative Interdisciplinary Research on Human-Nature Relationships

**DOI:** 10.3389/fpsyg.2022.809009

**Published:** 2022-04-06

**Authors:** Patricia Masterson-Algar, Stuart R. Jenkins, Gill Windle, Elisabeth Morris-Webb, Camila K. Takahashi, Trys Burke, Isabel Rosa, Aline S. Martinez, Emanuela B. Torres-Mattos, Renzo Taddei, Val Morrison, Paula Kasten, Lucy Bryning, Nara R. Cruz de Oliveira, Leandra R. Gonçalves, Martin W. Skov, Ceri Beynon-Davies, Janaina Bumbeer, Paulo H. N. Saldiva, Eliseth Leão, Ronaldo A. Christofoletti

**Affiliations:** ^1^School of Medical and Health Sciences, Bangor University, Bangor, United Kingdom; ^2^School of Ocean Sciences, Bangor University, Bangor, United Kingdom; ^3^SOS Mata Atlântica Foundation, São Paulo, Brazil; ^4^School of Natural Sciences, Bangor University, Bangor, United Kingdom; ^5^Institute of Marine Sciences, Federal University of São Paulo, Santos, Brazil; ^6^Institute of Health and Society, Federal University of São Paulo, Santos, Brazil; ^7^School of Human and Behavioural Sciences, Bangor University, Bangor, United Kingdom; ^8^College of Human Sciences, Bangor University, Bangor, United Kingdom; ^9^Natural Resources Wales, Bangor, United Kingdom; ^10^Boticario Group Foundation, São Paulo, Brazil; ^11^Faculty of Medicine, University of São Paulo, São Paulo, Brazil; ^12^Albert Einstein Israelite Hospital, São Paulo, Brazil

**Keywords:** interdisciplinary, global agendas, co-design, One Health, Ocean Decade

## Abstract

Strong evidence shows that exposure and engagement with the natural world not only improve human wellbeing but can also help promote environmentally friendly behaviors. Human-nature relationships are at the heart of global agendas promoted by international organizations including the World Health Organization’s (WHO) “One Health” and the United Nations (UN) “Ocean Decade.” These agendas demand collaborative multisector interdisciplinary efforts at local, national, and global levels. However, while global agendas highlight global goals for a sustainable world, developing science that directly addresses these agendas from design through to delivery and outputs does not come without its challenges. In this article, we present the outcomes of international meetings between researchers, stakeholders, and policymakers from the United Kingdom and Brazil. We propose a model for interdisciplinary work under such global agendas, particularly the interface between One Health and the UN Ocean Decade and identify three priority research areas closely linked to each other: human-nature connection, conservation-human behavior, and implementation strategies (bringing stakeholders together). We also discuss a number of recommendations for moving forward.

## Introduction

Linking science to decision making to achieve societal benefits can present a challenge for scientists, policy makers, and practitioners. Identifying research gaps, implementation barriers, and priorities linked to global agendas offers a pathway to address such challenges promoting socially relevant science and providing insights from knowledge to action. The World Health Organization (WHO) and the United Nations (UN) use the relationship between humans and their surrounding natural world as a fundamental pillar of their global agendas. Such agendas demand interdisciplinary and multisector collaborative efforts at a local, national, and global level. Improving and strengthening such efforts are crucial to support and achieve agenda goals.

The One Health approach aims to improve the understanding of how the health of organisms, people, and the environment are intimately connected (Lee and Brumme, 2013). This vision (Murray et al., 2014) has recently been pushed to the top of the agenda due to the current global health and environmental challenges, including the COVID-19 pandemic (Souza et al., 2021). There has been substantial investment by a number of funding organizations and governments (Defra, 2018) to enhance collaboration across human, animal, and environmental health sectors (Mazet et al., 2014; Khan et al., 2018). For example, the United Kingdom government has set out to use natural outdoor spaces (green spaces) and rivers, lakes, and coastal waters (blue spaces) to improve the health and wellbeing of its population. Alongside a plan to create and conserve outdoor spaces, they have set out to develop programs to encourage closeness to nature, with particular focus on disadvantaged areas and the negative impacts of loneliness and social isolation [Department for Environment, Food & Rural Affairs (DEFRA), 2018]. Interdisciplinary research has also been influenced by the UN declaration of 2021–2030 as the Decade on Ecosystem Restoration all closely linked to the Sustainable Development Goals (SDG) of the Agenda 2030 (Ryabinin et al., 2019; Fischer et al., 2020). While global agendas summarize multi-sectoral discussions and goals for a sustainable world, science is required to link across these agendas, from conceptualization and design through to outcomes and impact.

Working across natural, social, and health sciences can be a way forward to address these agendas. Policies and practices that nurture our environment while being mindful of societal needs should promote care, protection, and sustainability for both the planet and people from all backgrounds (Kelly, 2018). This requires us to review how we produce science, including our scientific principles, aims, and methods and the way in which we balance the implementation of top-down and bottom-up approaches (Pereira et al., 2019, 2021). Additionally, the way we spread and disseminate the usable knowledge has to be adjusted through networks to align science to social needs (Phelps et al., 2012). It also needs to accept the increasingly significant role that social media plays in eliciting responses from the public and hence, its potential impact through influences on decision makers. However, doing so presents many challenges. An integrated approach to research that is directly linked to global agendas is often discussed and desired, but exchange of knowledge among disciplines and stakeholders is a challenge, not only in sciences but also in politics and the private sector (Chien, 2013; Spencer et al., 2019). Collaborative approaches urgently need strengthening if we are to succeed in moving from concept to practice. The transformative science concept for the UN Ocean Decade provides a clear pathway for a change in science, based on co-design, solution-focused approaches open, and accessible to all and integrating generational, gender, and geographic diversity, including local and indigenous knowledge (UNESCO, 2021). Furthermore, evidence shows that there is a need for better monitoring, evaluation, and measuring of outcomes of this type of interdisciplinary approach which would support the case for future funding and would support the development of guidelines and best practice (Errecaborde et al., 2019; Spencer et al., 2019). For example, more needs to be done to firstly, evaluate the effectiveness of blue/green space therapeutic interventions on physical and mental wellbeing. Secondly, to determine the factors that are most effective in promoting different health and wellbeing outcomes, and finally, to understand the relationship between blue/green spaces, coastal proximity, health, and wellbeing, which can support environmental management and planning decision making (Gascon et al., 2017; Garrett et al., 2019).

## Working Across Sectors and Disciplines

This article reports on the outcomes of a 3-day international workshop with researchers, stakeholders, and policymakers of both the United Kingdom and Brazil. Our goal was to explore how to produce a working model across countries and disciplines that addresses global agendas, particularly the interface between One Health and the UN Ocean Decade. Brazil, like all countries on the Development Assistant Committee list, is facing significant challenges in health and in conservation of its diverse environments. Non-communicable diseases (including cancers, dementia, diabetes, and mental illness) accounted for 74% of premature deaths in Brazil (WHO, 2018a,b), while 47% of the adult population are thought to be at increased risk of premature death due to lack of physical activity (Beltrán-Sánchez and Andrade, 2016; Herazo-Beltrán et al., 2017). Equally, its coastline suffers many problems associated with rapid urbanization including habitat loss and pollution. For instance, contamination of coastal waters by untreated domestic sewage is a major environmental problem (Martinez et al., 2021) with negative impacts on the environment (Arevalo et al., 2007; Wear and Thurber, 2015) and health of coastal communities (e.g., respiratory infections, gastroenteritis, and hepatitis A; Shuval, 2003). Additionally, the very complex nature of the environmental problems that this region faces demands institutions to work as part of a team to create flexible, adaptive, and fit-for-purpose policies to secure social-ecological justice and wellbeing for all humans (Gonçalves et al., 2020a).

Brazilian policy makers recognize the link between public health and the environment and have set as a priority the need for cities across Brazil to engage with and support interdisciplinary research that can inform relevant environmental, planning, and public health policies. However, Brazil is currently lacking evidence base and interdisciplinary research capacity in order to develop and evaluate initiatives that will put this into action. In nations like the United Kingdom, there is a significant disconnect between increased urbanization and nature conservation resulting in the wellbeing and health benefits of the environment becoming increasingly out of reach (Gittins et al., 2021). Also, to date, the environment is largely “untapped” as a resource that can moderate rising health and wellbeing issues (Brink et al., 2016). Hence, the workshop aims were in line with three Sustainable Development Goals (SDGs): SDG11 (Sustainable Cities and Communities), SDG3 (Good Health and Wellbeing), and SDG10 (Reduced Inequality). Discussions during the workshop integrated these SDGs as opposed to looking at them in isolation. The workshop took place online in May 2021 with a total of 24 participants (13 based in Brazil and 11 in the United Kingdom) from academia, non-governmental organizations (NGOs), and governmental sector working across a wide range of areas, such as psychology, anthropology, health, physical education, ocean and marine sciences, conservation, and biological sciences.

**Figure 1:**
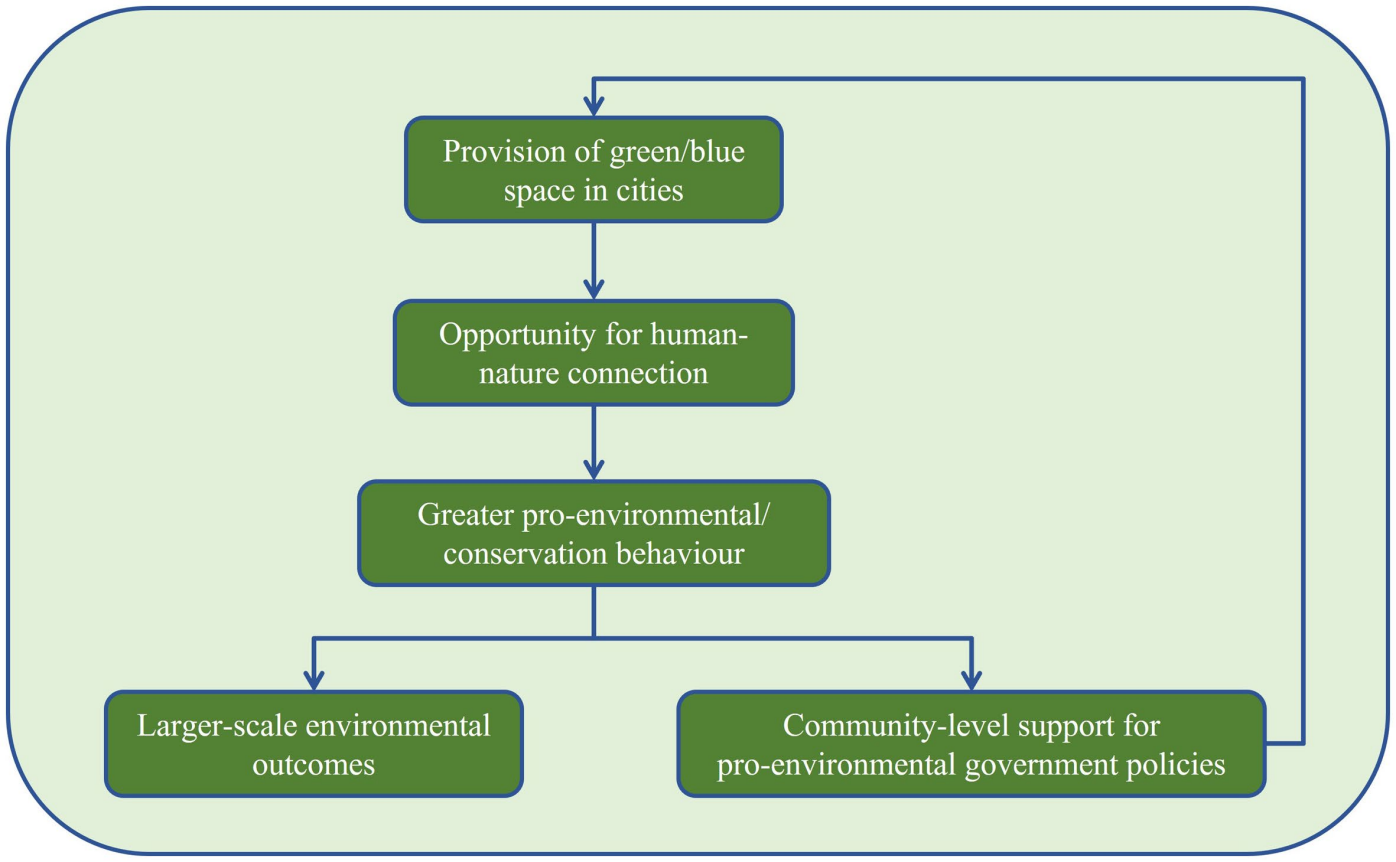
Conceptual model linking the provision of green/blue spaces with community-level support for pro-environmental government policies.

During the workshop, participants first identified research interests within disciplines in each country, followed by a discussion between countries for both health and environmental sciences. In sequence, working in mixed groups, participants identified links between their work and the two global agendas, One Health and UN Ocean Decade. Finally, participants engaged in in-depth discussions to identify research questions that integrated their work and also contributed to these agendas. The meetings were recorded and subsequent thematic analysis (Braun and Clarke, 2006) of recordings resulted in the identification of three interdisciplinary priority working areas to address these global agendas (Table 1). Informed by these areas, we were able to propose a conceptual model which represents a potential causal pathway linking the provision of green/blue spaces with community-level support for pro-environmental government policies (Figure 1). Future work will focus on evaluating this pathway as well as identifying new ones. We define our three identified priority areas as:

**Table 1:** Interdisciplinary priority areas for action which address the One Health and United Nations (UN) Ocean Decade agendas.

Human-nature connections	Conservation-human behavior	Implementation
Socioeconomic barriers Impact on the multiple dimensions of human health and wellbeing Relationship between urban green and blue spaces and improved health (healthy behaviours) Research on “Healthy Cities” and potential comparisons between rural/urban contexts	Impact of resource overexploitation and other human activities on the environment and hence on the livelihoods/wellbeing of communities The role of urban green infrastructure in mitigating the impact of threats such as climate change Identification of synergies and trade-offs between both, human wellbeing and conservation objectives	Bottom-up approaches at a community level—including production of tailored toolkits to inform planning, health, and conservation policies Creation of working links with local government Development of clear communication strategies in different contexts (e.g., education and business sector)

### Human-Nature Connections

Human-nature connection focuses on increasing our understanding of the magnitude of the connection between humans and their natural environment and the mechanisms (e.g., personal, sociocultural, economic, infrastructural, and political) driving it. Such connectedness brings benefits to both humans and nature (Maretti et al., 2019), improving human wellbeing while promoting pro-environmental behaviors (McEwan et al., 2019; Pritchard et al., 2019; Richardson et al., 2020). Surveys in England found that people who chose to have contact with nature more than once per week were more likely to improve their general health and also engage in pro-environmental behaviors, such as recycling, green transport, and encouraging others to protect the environment (Martin et al., 2020). Gittins et al. (2021) showed wellbeing gains from time spent in the woods (Actif Woods Wales program) for people with a range of health and social vulnerabilities. Fairchild et al. (2018) examined how greater habitat diversity can play a role in enhancing human interest in conservation, facilitating educational and recreational benefits. This working area focuses on identifying, within the context of both United Kingdom and Brazil, socioeconomic barriers to this human-nature connection which often lead to unequal access to outdoor spaces. There is a growing urgency for work to create “Healthy Cities” (WHO, 2012) that improve health outcomes for inhabitants (Gittins et al., 2021; Rice, 2021). Cities such as Santos (population: 430,000) or São Paulo (population: 12 million) can act as case studies to not only investigate the concept of “Healthy Cities” but also to explore potential comparisons between coastal/rural/urban-United Kingdom/Brazil contexts. With those case studies, it is possible to assess multiple impacts on health and wellbeing through a comparative research agenda aimed at the interface between metropolitan and coastal areas in places of intense urbanization. As our proposed conceptual model shows (Figure 1), this can encourage and stimulate a move toward coastal and metropolitan sustainability in a context of cross-scale environmental governance (Gonçalves et al., 2020b).

This area is informed by strong evidence that supports (a) the relationship between urban green spaces and improved mental health, frequently linked to the uptake of healthy behaviors including physical activity [Richardson et al., 2020; NERC (Valuing Nature Programme), 2021], (b) that urban green space interventions are most effective when coupled with social engagement and health outcomes that reach out to new target groups across all demographics (WHO, 2017), and (c) coastal zones (and other blue spaces) provide varied benefits that increase the health and human quality of life (Souza-Araujo et al., 2021). Work in this area requires radical approaches which embrace alternative epistemologies, local “expert” knowledge and societal/behavior change. The sense of belonging to nature and the respect that comes from the awareness of this integration must be the basis of a sustainable relationship between human beings and nature (Moore and Milkoreit, 2020). Science also needs to advance in investigating and providing evidence not only about understanding this interaction and its personal and societal outcomes, but also how to improve it.

### Conservation-Human Behavior

Conservation-human behavior focuses on addressing the need for an increased evidence base on the impact of resource overexploitation and other human activities on the environment and hence on the livelihoods and wellbeing of communities in order to promote, nurture, and manage these activities and relationships in the future. Understanding why people behave the way they do, at the personal level but also in institutional and corporate settings, is a key to managing these human behaviors and conserving the environment for future generations. This includes exploring how human-nature interactions may vary across scales (local to global) and over time (short to long-term impacts), and what drives these interactions. This area works on the premise that by valuing and creating an attachment with nature, through increased personal wellbeing, we can potentially increase sustainable attitudes. Nature connectedness has been found to be positively related to wellbeing, household pro-environmental behaviors (such as recycling, green transport, and encouraging others to protect the environment), and nature conservation behaviors (such as volunteering to help care for their environment; Martin et al., 2020). Thus, the emotional meaning people feel through interacting with nature can be harnessed for greater conservation efforts.

Understanding human behavior and social practices in a conservation context focuses on developing and evaluating human behavior driven initiatives to reduce and monitor the impact that increased urbanization is having on the green and blue spaces of cities such as Santos. It draws on research that demonstrates that innovative urban green infrastructure, such as protected areas and urban parks, can help mitigate the impact of climate change while supporting human and animal wellbeing (Pinto et al., 2020) but only when work is carried out across sectors open to identify common human and conservation objectives (Hassen, 2016; Hoyle and Gomes Sant’Anna, 2020). Ultimately, framing nature as a therapeutic tool has the potential to simultaneously meet human and conservation needs thus allowing for inclusive policy decision making, such as developing bespoke outdoor spaces in cities (Kelly, 2018). However, in developing policy and implementing specific actions, it is necessary to recognize and guard against the potential for the growing number of people accessing green and blue spaces for health benefits, to threaten biodiversity, and the integrity of ecosystems. This is because natural spaces are inevitably modified to accommodate human use. “People-friendly” spaces are not necessarily “wildlife-friendly.” Hence, we need a broader approach that aims to balance conservation principles with the need for esthetic features or maximizing activities in natural environments (Shanahan et al., 2019).

### Implementation (Co-design and Co-delivery)

Implementation (co-design and co-delivery) focuses on optimizing successful implementation of evidence generated in areas 1 and 2 and on the vital role of effective and accessible communication strategies (aiming at raising awareness, education, and engagement) in improving human-nature relationships and reducing health inequalities. This is based on the premise that local governments, through leadership, can play a vital role in implementation success which in turn can lead to addressing the health divide (WHO, 2012). Aspects related to safety, accessibility, and access to nature, especially for economically disadvantaged and socially vulnerable populations should also be part of this scenario. This area of work will require active engagement of key stakeholders and the application of bottom-up approaches at a community level to develop (or tailor) tools that can inform and transform both conservation and health policies. There is increasing attention on economic evidence of environmental health policy and health benefits from natural environments (WHO, 2013). Funding for policy change or community programs needs to be evidence-based and justified to enable successful implementation. Improvements in health and wellbeing from environmental interventions can also have wider social and economic gains including saving on healthcare costs from a reduction in chronic health conditions (Lynch et al., 2020). Finally, considering a wide range of stakeholders is important in co-producing change and in driving recognition of the societal value of green and blue spaces. Such understanding of the benefits to local and national economies is necessary to evidence the case for investment in environmental infrastructure (Lynch et al., 2020).

## Moving Forward—Recommendations

As a result of the workshop, six recommendations on how to conduct research following the One Health approach and goals of the UN Ocean Decade were suggested. The group identified that there is a need:

- To further develop mixed methods and participatory methodologies, i.e., research designs that incorporate innovative quantitative and qualitative approaches to generate in-depth understanding of human experiences, the environment, and wider systems.
- To better “exploit” available datasets and published evidence. This requires more open-access availability and investment in adequate training and guidance, but also more academic awareness of data held by public bodies (such as the *Monitor of Engagement with the Natural Environment survey*, Martin et al., 2020). More robust research designs for a higher level of evidence are also needed.
- For mutual learning between natural, social, and health sciences. This is necessary to understand the interlinkages between the fields to facilitate research which generates usable knowledge leading to action and social transformation.
- To embed theoretical frameworks into the design and development of research studies. Theories of human behavior, of resilience, and of social inclusion were identified as critical components to better understand human-nature-health connections.
- To promote bottom-up approaches that include co-production and co-design in order to explain the reasons that drive people to interact and benefit from their environments.
- To acknowledge that multidisciplinary knowledge and projects co-designed with stakeholders can strengthen sustainability through holistic governance systems, allowing changes of institutions, values, and practices.

## Conclusion

The workshop and the creation of this network have proven that it is possible to bring together researchers, stakeholders, and policymakers from different backgrounds working toward science linked to global agendas. It showed that global agendas can become a bridge that links a wide range of disciplines and that together we can engage in scientific research that addresses the intertwined nature of current societal challenges. Also that it is possible to accommodate socioeconomic-cultural differences among countries by strengthening co-design and mutual learning. As a result, a United Kingdom/Brazil network was created. To promote scientific and socially impactful research, we need efforts that are interdisciplinary, based on scientific evidence and that cut across sectors and cultures. We also need to address and exploit the increasingly vital role of digital dissemination pathways, such as social media, in generating impact. These efforts are vital to have a chance to successfully put into action the global health and environmental agendas for the next decades. We would like this paper to play a role in encouraging the creation of other networks and to promote communication between them.

## Data Availability Statement

The raw data supporting the conclusions of this article will be made available by the authors, without undue reservation.

## Author Contributions

PM-A led the funding acquisition and the workshops, carried out data analysis, and wrote the original draft. SJ, GW, and RC shared senior authorship and have contributed to the writing and editing of the original draft and with funding acquisition. All authors contributed to the article and approved the submitted version.

## Funding

This work was financially supported by: Bangor University Global Challenges Research Fund; São Paulo Research Foundation (FAPESP) grants #2017/50220-8, 14/50848-9, 15/50687-8 and 2016/11947-7; British Council—Newton Fund Grant Agreement Institutional Links #332425662; and Brazilian National Council for Scientific and Technological Development (CNPq), grant #434706/2018-3.

## Conflict of Interest

The authors declare that the research was conducted in the absence of any commercial or financial relationships that could be construed as a potential conflict of interest.

## Publisher’s Note

All claims expressed in this article are solely those of the authors and do not necessarily represent those of their affiliated organizations, or those of the publisher, the editors and the reviewers. Any product that may be evaluated in this article, or claim that may be made by its manufacturer, is not guaranteed or endorsed by the publisher.
